# Digital Therapeutics for Cognitive Impairment: Exploring Innovations, Challenges, and Future Prospects

**DOI:** 10.2196/73689

**Published:** 2025-06-12

**Authors:** Grace Yuange Zang, Keqin Rao, Jing Wu, Yunhan He, Yi Tang, Leiyu Shi

**Affiliations:** 1 Department of Health Policy and Management, Bloomberg School of Public Health, Johns Hopkins University, Baltimore, MD, United States; 2 Institute for Hospital Management, Tsinghua University, Beijing, China; 3 National Center for Chronic and Noncommunicable Disease Control and Prevention, Chinese Center For Disease Control and Prevention, Beijing, China; 4 School of Psychology, Shenzhen University, Shenzhen, China; 5 Department of Neurology & Innovation Center for Neurological Disorders, Xuanwu Hospital, Capital Medical University, National Center for Neurological Disorders, 45 Changchun Street, Beijing, 100053, China, 86 13811021432

**Keywords:** digital therapeutics, cognitive digital therapeutics, cognitive impairment, innovative applications, health technology, artificial intelligence, multistakeholder collaboration

## Abstract

Recent advancements in cognitive neuroscience and digital technology have significantly accelerated the adoption of digital therapeutics for cognitive impairment. This viewpoint explores the innovative applications of digital therapeutics in the assessment, intervention, management, and monitoring of cognitive disorders while highlighting key challenges that impede their widespread integration into clinical practice. Drawing on the definition of cognitive digital therapeutics (CDTx) and the multistakeholder collaboration required for its development and implementation, this paper examines the role of digital technologies in cognitive health and explores challenges from multiple perspectives, including clinical practice, policy framework, user adoption, ethics and privacy, and data interoperability and system integration. In addition, this viewpoint offers strategic recommendations to address the challenges and future prospects of CDTx, emphasizing the importance of multistakeholder collaboration, prioritizing user-centered design, and leveraging emerging technologies such as artificial intelligence to enhance the scalability, sustainability, and future integration of CDTx.

## Introduction

Cognitive impairment is a transitional stage between normal age-associated cognitive decline and dementia. Cognitive impairment encompasses a broad spectrum of syndromes characterized by persistent deterioration in cognitive function, which ultimately leads to a decline in daily living and working abilities, along with behavioral changes, placing a substantial burden on patients, families, and society. Globally, 43.8 million people were living with Alzheimer disease and related dementias in 2016, and this number may increase to 152 million by 2025 [1], with the associated economic burden estimated at US $2.8 trillion in 2019 and projected to exceed US $4.7 trillion by 2030 [2]. As the condition progresses, patients increasingly rely on informal caregivers, who face not only physical and emotional stress but also financial strain, including reduced work hours and income loss [3,4]. These trends underscore an urgent need for scalable, innovative, and cost-effective solutions to support individuals with cognitive impairment and their caregivers.

Advancements in information technology have paved the way for innovative medical technologies, supporting clinical practice, enhancing disease prevention, and improving prognostic management, offering significant potential in health care [5]. Since the establishment of the Digital Therapeutics Alliance in 2017, digitally-based therapeutic methods, digital therapeutics (DTx), have been widely researched and applied. DTx deliver medical interventions directly to patients using evidence-based, clinically evaluated software to treat, manage, and prevent a broad spectrum of diseases and disorders [6,7]. Currently, there is no universally accepted definition of DTx at the international level, however, DTx solutions commonly share key characteristics, including the use of high-quality software, rigorous evidence-based validation, and targeted therapeutic interventions [8,9]. DTx have the potential to reduce health care costs, save diagnostic and treatment expenses, improve disease diagnosis and treatment efficiency, increase patient accessibility, optimize disease treatment and management plans, enhance patient adherence, and improve treatment outcomes [4].

Cognitive digital therapeutics (CDTx) refer to innovative applications of DTx in the field of cognitive disorders. These software-driven interventions provide patients with evidence-based digital solutions for cognitive assessment, prevention, treatment, and ongoing management. Powered by clinically validated software programs, CDTx offer evidence-based approaches for cognitive assessment, behavioral intervention, and monitoring and management of cognitive disorders, providing scalable and personalized solutions to support cognitive care across diverse care settings. Despite their promise, the adoption of CDTx remains uneven and fraught with real-world challenges, particularly in reaching older populations, navigating regulatory ambiguity, ensuring data privacy, and achieving effective clinical integration. While recent literature offers valuable insights into the clinical efficacy and application scenarios of specific CDTx, ranging from randomized controlled trials of cognitive training for older adults [10], home-based digital interventions for patients with Alzheimer disease (AD) [11], and meta-analyses of computerized cognitive training [12], to reviews on digital support for patients, caregivers, and health care professionals [13], and regional digital cognitive screening programs for early prevention in developing settings [14], research that integrates technological innovation with the practical realities of cognitive care remains limited. Most existing studies focus on isolated interventions, lacking a structured perspective that addresses both the challenges and prospects of implementing DTx in this field. This absence of a comprehensive perspective leaves a critical gap in understanding how to scale innovation, address real-world barriers, and fully realize the potential of CDTx in routine clinical practice.

This study aims to explore the current landscape of CDTx by synthesizing published literature and our clinical experiences on recent innovations and critically assessing the challenges that limit their broader integration into health care systems. Through this perspective, we aim to provide timely, actionable insights for practitioners, researchers, and policy makers working to advance cognitive care. Ultimately, we underscore the potential of DTx to promote healthy aging and improve cognitive outcomes for aging populations worldwide. This study is structured around the following key dimensions: the innovative applications of CDTx, the emerging challenges, and the future prospects in their real-world adoption. Specifically, we explore how CDTx are currently applied in cognitive assessment, intervention, and long-term management across health care settings. We then analyze major challenges, such as limited clinical evidence, inadequate regulatory and policy frameworks, user engagement issues, ethical and privacy concerns, and technical interoperability, that constrain broader implementation. Finally, we highlight strategic prospects to support scalable, sustainable, and equitable integration of CDTx into routine care.

## Innovative Applications of DTx for Cognitive Impairment

### Digital Cognitive Assessment

#### Overview

Traditional paper-and-pencil cognitive tests, such as the Mini-Mental State Examination and the Montreal Cognitive Assessment, are widely used in clinical practice. However, these conventional methods exhibit numerous limitations, including the necessity for professional personnel to conduct face-to-face interviews, the potential for human bias in test results, and practice effects resulting from repeated administrations [15,16]. To overcome these limitations and support more scalable approaches to assessment and screening, digital cognitive assessments have emerged, leveraging emerging technologies to achieve automated, standardized, convenient, multidimensional evaluations of cognitive functions. Over the past 2 decades, digital cognitive assessment has experienced rapid development, with extensive research and adoption in clinical settings [15,16]. These digital assessments rely on objective, quantifiable, physiological, and behavioral data collected by digital devices, enabling continuous ecological assessment and long-term monitoring of cognitive health. Many CDTx solutions integrate assessment features, such as digital assessment and screening tests, to track cognitive status over time. Based on functionality, technological characteristics, and application scenarios, digital cognitive assessment can be broadly categorized into three primary types: (1) computerized cognitive assessment, (2) digital biomarker-driven cognitive assessment, and (3) multimodal brain imaging cognitive assessment.

#### Computerized Cognitive Assessment

Computerized cognitive assessments include both scale-based assessments and task-based assessments, developed based on traditional pencil-and-paper tests, which have been viewed as the gold standard for diagnosis. For example, digitalized versions of the Montreal Cognitive Assessment, Mini-Mental State Examination, and Alzheimer’s Disease Assessment Scale-Cognitive Subscale have been validated and show good consistency with traditional paper-and-pencil methods [17], offering advantages such as automated scoring and real-time data analysis. Meanwhile, task-based assessments like the Cambridge Neuropsychological Test Automated Battery [18], Brain-Check [19], Neurotrack cognitive Battery [20], and the Beijing Aging Brain Rejuvenation Initiative brain health system evaluate cognitive abilities such as memory, attention, and executive function through interactive tasks and simulated environments [21]. Furthermore, virtual reality (VR) environments and computerized cognitive games constitute significant components in this category. These digital technologies either simulate traditional cognitive assessments or create novel interactive tasks to evaluate a broad range of cognitive functions. For example, the Virtual Supermarket uses immersive environments to simulate shopping tasks, providing an effective method for assessing executive functions and memory capabilities [22]. In addition, computerized cognitive games, such as Episode Gamification, engage user engagement through gamified design while simultaneously evaluating various cognitive domains, including memory, attention, and executive functions [23].

#### Digital Biomarker-Driven Cognitive Assessment

Digital biomarkers are objective, quantifiable, physiological, and behavioral data collected and measured through digital devices, such as embedded environmental sensors, portable devices, and wearable. These data can be gathered in the context of daily activities with minimal intrusion, enabling ecological monitoring. Digital biomarkers are increasingly used for early detection and follow-up of cognitive impairment. For instance, older adults with cognitive impairments show significantly reduced daily activity ranges compared with cognitively normal older adults, with more restricted and stable activity patterns [24]. Therefore, monitoring their daily behavior trajectories can provide early warnings. Furthermore, by integrating machine learning and deep learning algorithms and incorporating speech and natural language processing from both healthy and patient populations, classifiers can be constructed to identify cognitive impairments. It has been found that speech fluency, complexity, and coherence can also serve as markers for early screening [25]. In addition, significant oculomotor impairments have been observed in cognitive impairment patients [26], which monitors eye movements and visual attention through tasks such as reading comprehension and visual search, providing insights into abnormal attention and visual behaviors [27].

#### Multimodal Brain Imaging Cognitive Assessment

Multimodal imaging techniques offer diverse perspectives for observing and analyzing the brain, providing critical imaging evidence for the early diagnosis of AD by assessing structural, functional, and metabolic changes. Currently, widely used multimodal imaging techniques for AD assessment include structural magnetic resonance imaging (sMRI), functional magnetic resonance imaging (fMRI), and positron emission tomography (PET) [28]. Additionally, diffusion tensor imaging (DTI) and other advanced methods provide critical insights into the disease's pathology and progression [28], and no imaging modality can serve all purposes as each has unique strengths and weaknesses [29]. In recent years, machine learning techniques have become increasingly integrated with multimodal imaging data, improving diagnostic efficiency and accuracy by classification and prediction. A widely used machine learning and deep learning approach in this context is ensemble learning, which integrates multiple machine learning models to optimize fusion strategies. For instance, Shukla et al [30] applied ensemble learning to positron emission tomography and T1-weighted MRI data from the Alzheimer Disease Neuroimaging Initiative dataset for both binary and multiclass classification of AD, cognitive impairment, and cognitively normal individuals. Their approach achieved up to 99% accuracy for binary classification and 96% for multiclass classification, demonstrating its potential in early AD diagnosis [30]. Convolutional neural networks have also shown strong performance in AD classification. Farooq et al [31] used deep convolutional neural networks with a 4-way classifier to categorize AD progression stages, achieving 98.8% accuracy on the Alzheimer Disease Neuroimaging Initiative dataset. Furthermore, neuromorphic computing, inspired by human brain mechanisms such as neural connectivity and synaptic plasticity, enhances both machine learning and deep learning models, excelling in cognitive function analysis and medical imaging tasks. In AD research, Turkson et al [32] demonstrated that spiking neural networks outperform traditional machine learning methods in predicting AD classification. In addition, a hybridization of machine learning and deep learning is also used for classifying AD, achieving an accuracy of 91.84% on test data [33].

Digital cognitive assessment methods offer distinct functional strengths across modalities, and their thoughtful integration may provide a more comprehensive understanding and assessment of cognitive performance in real-world environments and clinical settings.

### Digital Cognitive Intervention

#### Overview

There is currently no definitive cure or intervention for cognitive impairment, with existing management strategies primarily aimed at symptom control and slowing disease progression [22]. In parallel, pharmaceutical approaches face significant challenges, with AD drug development showing a failure rate as high as 99.6% [34]. Given the lack of an intervention for cognitive impairment and dementia, research has increasingly focused on delaying disease progression and developing interventions to enhance and preserve cognitive function [35]. CDTx have emerged as a promising direction in this context, particularly for older adults with cognitive impairment. These interventions can be broadly categorized into 3 types. The first involves strengthening existing cognitive pathways through behavioral exercises to enhance specific cognitive functions; the second targets the direct modulation of brain activity to improve overall brain function or the targeted brain regions; and the third aims to identify and manage risk factors associated with cognitive decline, such as lifestyle behaviors, chronic diseases, and mental health issues, with the goal of mitigating root causes and delaying onset.

#### Behavioral Training for Cognitive Enhancement

Many traditional cognitive interventions have been adapted for use on current technological devices, such as smartphones, tablets, and computers, and corresponding digital interventions have been developed based on identified evaluation metrics. These approaches are not only cost-effective alternatives but also innovative solutions for cognitive enhancement [36]. For instance, CogniFit (CogniFit Ltd) implements personalized and gamified cognitive tasks that participants can perform at home. This intervention effectively enhances cognitive function while promoting engagement and adherence through psychoeducation and behavior modification techniques. It has been shown to significantly improve cognitive impairments in older adults [37]. Furthermore, video games combining cognitive gameplay with physical exercise, referred to as “Exergames,” have been developed. These games have been shown to improve physical function, reduce depression, and enhance cognition and quality of life in older adults [38]. VR also represents a promising intervention, with its immersive and interactive features making it well-suited for simulating complex environments and tasks to improve cognitive training outcomes. Although studies validating the effectiveness of VR remain relatively limited, existing research suggests its potential to improve memory function and social interaction [39]. Further research is needed to optimize the design and broaden the application of these technology-based interventions [40].

#### Direct Modulation of Brain Activity for Cognitive Improvement

This approach primarily uses neuro-modulation technologies to target specific brain regions or neural circuits, enhancing or restoring brain function. Repetitive transcranial magnetic stimulation is a widely used technique that has been shown to improve cognitive function in patients with cognitive impairment [41], and various combinations of repetitive transcranial magnetic stimulation parameters have been found effective in enhancing different cognitive domains–based approaches suggest that cognitive impairments are linked to distributed brain networks rather than isolated regions [42]. The corticohippocampal circuit, crucial for long-term memory, can be targeted through paired associative stimulation (PAS) to enhance memory connections. Studies have shown that PAS improves delayed recall in cognitive impairment by synchronizing neuronal activity within this network [43]. In addition, PAS mimics sleep-related neural oscillations, facilitating memory consolidation and optimizing memory training outcomes [44].

#### Identification and Intervention of Risk Factors for Cognitive Decline

The 2024 Lancet Commission reports that around 45% of dementia cases may be prevented or delayed by addressing 14 modifiable risk factors, including lifestyle, health, environmental, and social factors [45]. In line with this, another approach to improving cognitive abilities involves addressing risk factors that impair cognitive development, such as diabetes, depression, and hypertension [46]. For example, Deprexis, a digital tool based on psychotherapy, helps improve mood and increase positive emotions and behaviors [47]. The HERB Digital Hypertension 1 (HERB-DH1) alleviates hypertension by offering education and lifestyle guidance, including reducing salt intake, controlling weight, exercising, improving sleep quality, and managing stress [48]. In addition, other digital intervention tools targeting diet, smoking, and alcohol consumption can also be effectively used to address these risk factors.

Digital cognitive interventions reflect an evolving understanding that cognitive health is related not only to neural mechanisms, but also to behavioral, psychological, and environmental factors. As such, digital cognitive interventions hold significant potential to complement existing care models and broaden access to timely, personalized support.

### Digital Cognitive Management and Monitoring

#### Overview

Cognitive management and monitoring refer to the ongoing tracking of cognitive functions over time, offering a longitudinal view of an individual’s cognitive health. This approach captures fluctuations in cognitive abilities, supports early detection of decline, and enables timely intervention and cognitive care planning.

#### Cognitive Assistance Technology

Cognitive assistance technologies leverage digital tools, including information and communication devices and decision-support systems, to help individuals with cognitive impairment and their caregivers in daily cognitive care and management. These technologies aim to enhance quality of life, improve caregiving efficiency, and promote independent living. Personal digital assistance programs, such as smartphone apps, assist with daily life management by helping patients manage time, remember appointments, and organize tasks, which have been shown to significantly improve executive function in patients with acquired brain injuries [49]. In addition, many of these therapeutics integrate health monitoring features, such as medication reminders and physiological data tracking, providing valuable feedback loops for clinicians and care teams. Wearable devices such as smartwatches and fitness trackers further expand cognitive assistance by continuously monitoring physical activity, sleep patterns, and cognitive performance, enabling real-time adjustments to interventions as needed [50]. Multimedia-based tools, including video, audio, and internet-based assistive tools also provide rich resources for caregivers, including online training, remote video calls for timely support, and artificial intelligence (AI)–powered decision support systems that generate personalized care plans based on patients’ data [51].

#### Real-Time Cognitive Monitoring Technology

Real-time cognitive monitoring technologies collect objective physiological and behavioral data to continuously assess patients’ cognitive status and functional capacity in daily life. Devices such as actigraphy sensors, GPS, in-home cameras, and electroencephalography (EEG) systems enable and facilitate real-time tracking, analysis, and evaluation of cognitive status and intervention effectiveness. Mobile apps and mobile health solutions are increasingly used to provide task-based feedback and cognitive performance tracking over time, helping patients and clinicians identify patterns in cognitive function [52].

Advanced EEG methods, including quantitative EEG, are now being embedded into digital monitoring devices to detect abnormal brain activity and enable earlier identification of cognitive decline. Furthermore, machine learning algorithms and large language models applied to real-time data can forecast future cognitive decline based on behavioral and neural biomarkers, opening new possibilities for early diagnosis and proactive intervention [53].

Digital cognitive management and monitoring technologies mark a shift toward proactive, personalized, and data-driven cognitive care and management. By supporting both individuals and caregivers through real-time feedback and adaptive support systems, these tools offer meaningful opportunities to enhance quality of life and optimize care delivery across various stages of cognitive impairment.

**Figure 1.**
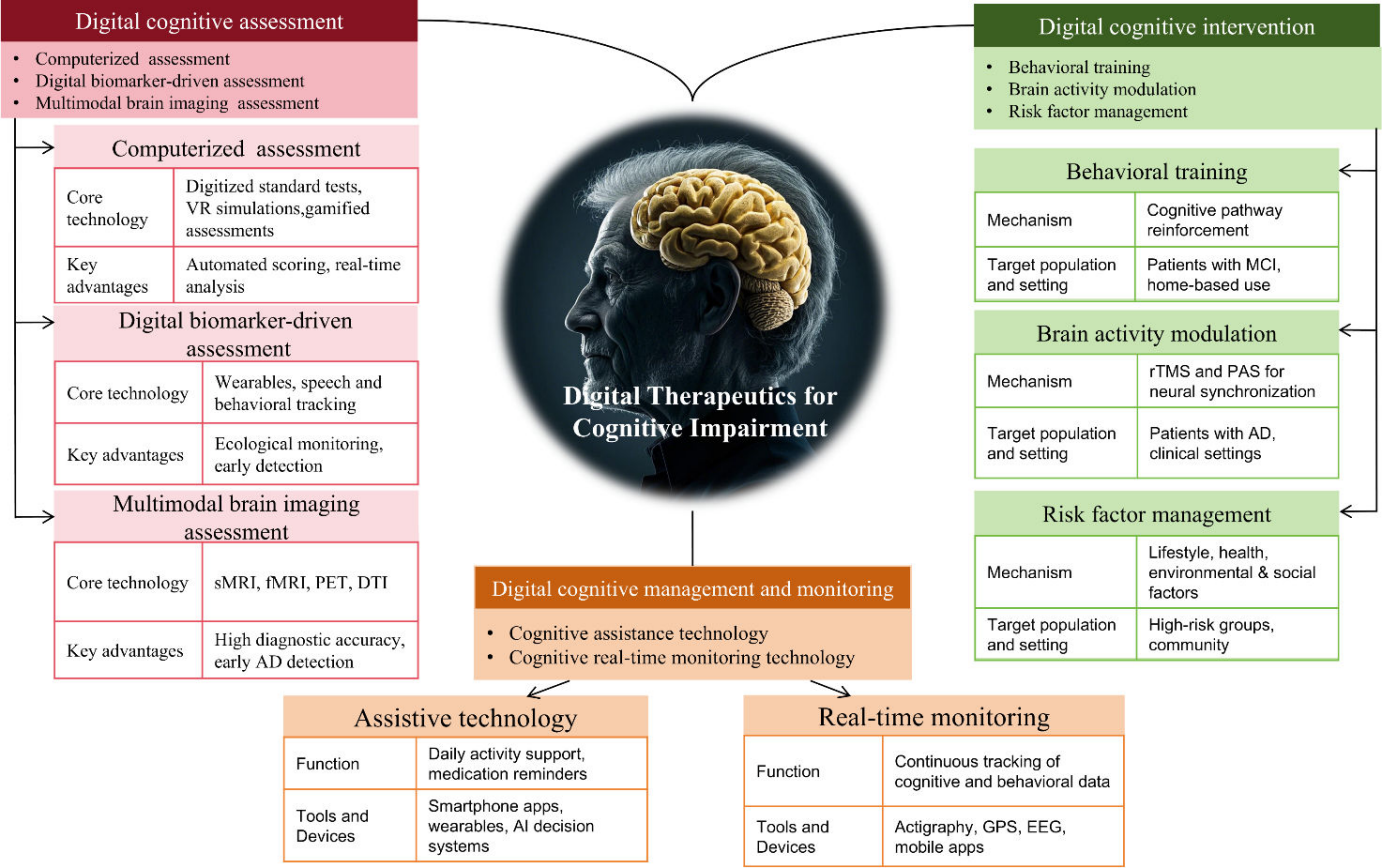
Cognitive digital therapeutics. AD: Alzheimer disease; AI: artificial intelligence; DTI: diffusion tensor imaging; EEG: electroencephalogram; fMRI: functional magnetic resonance imaging; MCI: mild cognitive impairment; MRI: magnetic resonance imaging; PAS: paired associative stimulation; PET: positron emission tomography; rTMS: repetitive transcranial magnetic simulation; sMRI: structural magnetic resonance imaging; VR: virtual reality.

Figure 1 presents a structured summary of the innovative applications of CDTx for cognitive impairment, categorizing their core functions into 3 domains—digital cognitive assessment, digital cognitive intervention, and digital cognitive management and monitoring. The figure highlights how these technologies are applied across the care continuum to support early detection, personalized treatment, and ongoing disease management for cognitive impairment.

## Challenges and Future Prospects of CDTx: Multistakeholder Perspective

### Clinical Evidence and Effectiveness

#### Challenges

CDTx must undergo rigorous clinical validation to ensure their adoption in health care. Despite growing enthusiasm, the lack of comprehensive clinical validation makes it difficult for stakeholders to assess their true value compared with conventional interventions. One major challenge lies in the heterogeneity of cognitive impairment populations, which complicates the development of standardized protocols and outcomes metrics [54]. This variability hampers the ability to draw consistent, generalizable conclusions from clinical trials and real-world implementations consistently.

Another critical obstacle is the rapidly evolving nature of DTx technologies, which often outpaces the development of clinical guidelines and regulatory frameworks. This technological dynamism can lead to discrepancies in trial designs, end point selection, and outcome measures, making comparisons across studies difficult and limiting the accumulation of robust evidence [55]. In addition, cultural and geographic variations in clinical practice and health care infrastructure further complicate the establishment of unified evidence frameworks that can be applied universally [56].

#### Future Prospects

To address these challenges, innovative strategies emphasize the potential of real-world evidence and home-based interventions. For example, remotely supervised programs that integrate both cognitive and physical training reduce patient and caregiver burden while enabling broader participation and scalable clinical data collection [57]. Likewise, leveraging data from electronic health records and wearable devices allows for the generation of real-world insights that complement traditional clinical trial findings, providing a more comprehensive understanding of DTx outcomes and performance [58].

Cross-sector collaboration is essential to accelerating clinical validation efforts. Consensus building among researchers, clinicians, and patient groups can help define standard outcomes and protocols, as well as the acceptable efficacy threshold [59]. International efforts and coordination on clinical trial methodology and data-sharing practices will be critical to facilitate evidence accumulation, consistency, and comparability of findings.

### Regulatory Uncertainty and Policy Gaps

#### Challenges

CDTx face significant regulatory challenges due to the absence of dedicated pathways for their development, validation, market entry, and postmarket oversight. Existing medical device regulations lack the flexibility required to accommodate rapidly evolving digital health technologies and DTx, leading to uncertainties in approval, compliance, and clinical adoption [60,61]. The lack of harmonized regulatory standards results in inconsistent prescribing criteria across jurisdictions, which complicates clinical integration and limits the scalability of DTx solutions [62,63]. In addition, ongoing debates over whether DTx should follow a prescription-based model or be available directly to consumers further fragment the regulatory landscape, influenced by divergent commercial strategies and legal frameworks [64].

Furthermore, current regulatory structures struggle to incorporate dynamic mechanisms that can keep pace with the fast-moving technological innovations [65]. Varying evidence requirements across countries or states often lead to redundant approval processes, increasing costs and delaying the timely entry of products into the market [66]. Furthermore, many current regulatory assessments and policies do not adequately account for the broader impact of DTx on patient education, behavioral change, and system efficiency [67], reflecting underlying policy gaps in recognizing the multifaceted value of DTx. Insufficient or absent postmarket surveillance mechanisms and inadequate policy oversight further limit the ability to monitor the long-term safety, real-world effectiveness, and scalability of CDTx [68].

#### Future Prospects

Several recent policy efforts suggest a shift toward greater alignment and innovation in regulating digital health. At the global level, leading regulatory bodies have also made strides in establishing pathways for the evaluation and approval of DTx. In the United States, the Food and Drug Administration adopted the Software as a Medical Device regulatory framework [9], which aligns with international guidelines developed by the International Medical Device Regulators Forum, this framework provides risk-based classification and guidance for DTx, with several products such as EndeavorRx, which is a digital therapeutic product designed by Akili Interactive, already approved under these standards [69]. In Europe, in its Regulatory Science Strategy to 2025, the European Medicines Agency proposes the creation of an integrated evaluation pathway for assessing medical devices, in vitro diagnostics, and borderline products including digital health technologies and therapeutics [70]. In China, guidelines from the National Health Commission, the National Administration of Traditional Chinese Medicine, and the Chinese Center for Disease Control and Prevention have laid the foundation for promoting AI applications and digital health technologies including DTx, providing a strategic framework and instructions for the application of AI and digital health in health care [71]. These guidelines emphasize the safe and strategic integration of emerging technologies into clinical pathways, providing a regulatory environment that supports both innovation and standardization.

Despite these advances, cross-national variations in evidence thresholds, regulatory timelines, and postmarket surveillance systems persist. This lack of harmonization and consistency often leads to duplicative regulatory burdens, increased costs, and slower global dissemination of DTx. To bridge the gap between technological innovation and outdated regulatory structures, tailored regulatory frameworks are needed to establish safety, efficacy, and usability standards while enabling adaptive to adjust and change that could accommodate ongoing technology advancements [72,73]. One promising model is the use of “regulatory sandboxes,” which provide controlled environments for testing DTx and digital health products under specific safety standards and regulatory oversight [73]. This approach enables more agile evaluation and iterative refinement, allowing regulatory bodies to monitor emerging therapeutics and technologies more closely, thereby accelerating time to market while safeguarding patient safety.

In addition, enhanced collaboration among regulators, policy makers, clinicians, and industry stakeholders will be essential to streamline prescribing guidelines and establish consistent approval criteria [65]. Global harmonization of clinical evidence requirements across jurisdictions can help reduce redundant approval processes, lower market entry barriers, and accelerate the adoption of DTx [66]. Finally, strengthening postmarket surveillance through real-time data analytics, patient registries, and adverse event tracking will enable regulators and policy makers to respond more dynamically, ensuring that DTx remain safe, effective, and sustainable over time [68].

### Patient Acceptance and Engagement

#### Challenges

The adoption and implementation of DTx depend significantly on user acceptance and long-term engagement, both of which are influenced by factors such as perceived usefulness, ease of use, trust, and accessibility. One persistent challenge is high dropout rates and nonadherence, often linked to difficulties in maintaining interest and motivation over time. For patients with cognitive impairments or limited digital literacy, these platforms may present a steep learning curve or misunderstanding, leading to user frustration, disengagement, and eventual abandonment of DTx. Another key issue is perceived usefulness, if users do not experience clear benefits such as improved health outcomes and enhanced quality of life compared with traditional treatment methods, they are unlikely to maintain consistent engagement [74]. Similarly, complex user interfaces, technical glitches, or insufficient support can act as further barriers to adoption [75]. These factors are especially important in older populations, where cognitive limitations may intersect with low technology familiarity.

Notably, a qualitative study involving older adults using a cognitive training DTx platform found that although some users experienced difficulties understanding the instructions, many still perceived the platform as both enjoyable and beneficial, particularly when it was recommended by health care providers. Participants expressed strong awareness of the importance of cognitive training and demonstrated greater trust and willingness to engage with digital health technologies and therapeutics that were clinician-endorsed [76].

#### Future Prospects

To address these engagement challenges, user-centered design must be placed at the core of DTx development. Iterative co-design approaches emphasize the importance of involving target users throughout the development cycle and can ensure that DTx products align with their real-world needs, preference, and capabilities [77]. Participatory design frameworks, such as those applied in the iReadMore app (Neurotherapeutics Group), have been shown to enhance usability and promote user motivation and accessibility through personalization and intuitive interfaces [78].

Tailoring DTx products to the cognitive and physical capabilities of target populations is critical. For example, interfaces designed with simplified navigation, voice support, and visual aids may increase accessibility for users with mild cognitive impairment. As Vial et al [79] discuss, human-centered and empathic design processes ensure that digital mental health interventions are both accessible and aligned with user needs, fostering trust and long-term usage. In parallel, trusted intermediaries, such as physicians, therapists, or community health workers, play a crucial role in increasing adoption by legitimizing the use of DTx and encouraging long-term use [79]. Wong et al [80] emphasize that user-centered design not only improves usability but also ensures integration with clinical workflows, enhancing the credibility of digital tools when endorsed by medical professionals. Furthermore, Strauss et al [81] argue that cross-functional frameworks, including input from patients and health care providers, result in solutions that are both effective and engaging. By addressing these barriers and emphasizing user-centered, humanized design, digital therapies can achieve greater acceptance and adherence among diverse patient populations.

### Data Privacy and Ethical Concerns

#### Challenges

The rapid development of DTx brings significant ethical and privacy-related concerns that must be addressed to ensure patient trust, data safety, and equitable access to care. One of the foremost challenges is data privacy and security, as DTx platforms often require the continuous collection and processing of highly sensitive personal health data. This introduces substantial risks of unauthorized access, data breaches, and misuse, particularly when robust data protection measures are currently lacking. These risks are further complicated by regional inconsistencies in data protection legislation, such as varying implementations of General Data Protection Regulation (GDPR)–like frameworks, leading to uneven security levels across jurisdictions [82].

Another critical concern is informed consent. Many patients, particularly those with cognitive impairments, struggle to fully understand the implications of data-sharing policies, especially when consent forms and data practices are opaque or overly complex, undermining the principle of truly informed consent [83].

In addition, digital health technologies frequently require reliable internet access, compatible devices, and basic digital literacy, creating barriers for underprivileged populations and potentially exacerbating health inequities. The lack of robust postmarket surveillance systems further compounds these concerns, as ongoing real-world safety and effectiveness data are often insufficient, making it difficult to oversee and identify emerging ethical risks [65].

#### Future Prospects

A comprehensive, ethically grounded strategy is essential to ensure the safe, ethical, and equitable deployment of CDTx. First, strengthening data protection frameworks is crucial, this includes mandating the use of encryption, anonymization, and secure data storage protocols, as well as enforcing compliance with international standards such as General Data Protection Regulation [84]. Second, simplifying consent processes and improving transparency can empower patients to make truly informed decisions [85], this may involve visual aids, plain language summaries, or interactive digital interfaces that clarify how personal data will be used. To further promote fairness, developers need to actively address algorithmic biases through inclusive design, regular ethical audits, and accountability mechanisms to ensure system decisions do not reinforce existing disparities [86,87].

Furthermore, integrating comprehensive postmarket surveillance on data privacy and safety as a core component of DTx regulation is vital, including real-time monitoring, longitudinal patient registries, and adverse event tracking and reporting systems to continuously monitor and enhance safety and ethical integrity over time [88]. Finally, ethically sound deployment of CDTx requires long-term collaboration among different stakeholders. Regulatory bodies, clinicians, industry developers, and patient advocacy groups must co-develop ethical guidelines and frameworks that reflect diverse values and lived experiences [89]. Such collaboration ensures that DTx are implemented in a way that protects individual data and rights while advancing clinical innovation.

### Data Interoperability and System Integration

#### Challenges

The effective integration of CDTx into existing health care systems is significantly hindered by issues of data interoperability and system integration. Seamless data exchange between CDTx platforms and electronic health records (EHRs) is crucial for clinical utility, yet technical fragmentation and inconsistent standards remain major obstacles [90]. Many health information technology systems operate in silos, and the lack of consistent technical standards across platforms, along with fragmented digital health ecosystems, poses significant barriers to building a unified infrastructure [91].

Furthermore, current health care infrastructures often lack the capacity to accommodate digital health and DTx products, especially in resource-limited settings [91]. Although standards such as HL7 Fast Healthcare Interoperability Resources (FHIR) are gaining momentum, implementation remains inconsistent and uneven across regions and health care organizations, leading to gaps in compatibility and functionality. These problems are compounded by the increasing reliance on internet of things devices, such as wearables and home monitoring tools, which often struggle to interface effectively with cloud-based EHR systems, this lack of compatibility limits the aggregation, accessibility, and clinical utility of patient data in clinical settings [92].

#### Future Prospects

Addressing these challenges requires the creation of an open, interoperable digital ecosystem. Adopting globally recognized interoperability standards will enhance the usability, integration, and scalability of CDTx [93,94]. Among these, HL7 FHIR has emerged as a foundational framework for standardized data exchange across diverse health care systems. Its modular architecture and open application programming interface design make it especially well-suited for supporting DTx in dynamic and data-intensive environments [95].

Beyond interoperability, data security must remain a parallel priority. Integrating blockchain technologies into FHIR-based systems has shown potential for enhancing the trustworthiness and traceability of shared health data while reducing risks of unauthorized access and data breaches [93].

In clinical practice, the successful integration of CDTx with EHR systems could unlock personalized decision support, reduce diagnostic errors, and enable more adaptive, data-driven care pathways [96,97]. As interoperability standards become more widely adopted and system integration capabilities continue to improve, DTx can evolve from standalone tools to fully embedded components within the cognitive care delivery system, significantly enhancing their effectiveness and sustainability.

**Figure 2.**
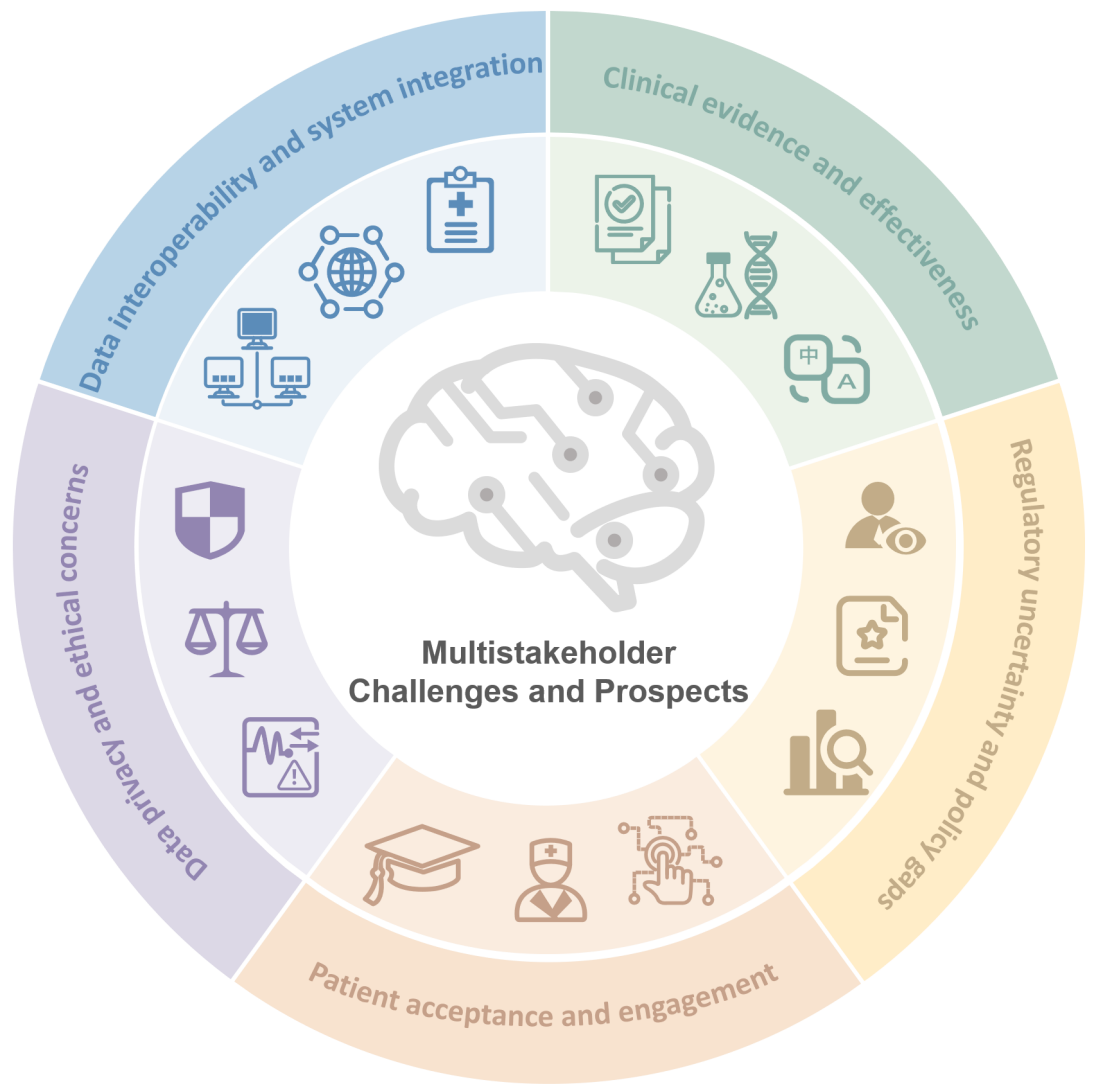
Summary of challenges and future prospects for cognitive digital therapeutics*.*

Challenges and future prospects of CDTx involve 5 key domains, that is, clinical evidence and effectiveness, regulatory uncertainty, patient acceptance, data privacy and ethics, and system interoperability. Figure 2 outlines these challenges and potential development pathways from a multistakeholder perspective. Table 1 complements this by summarizing key issues and proposed strategies across stakeholder groups, offering a practical foundation to support the clinical adoption and broader implementation of CDTx in cognitive care.

**Table 1.** Challenges, key issues, and future prospects for cognitive digital therapeutics.

Challenges	Key issues	Future prospects
Clinical evidence and effectiveness	Limited clinical validation Heterogeneous patient populations Lack of standard outcome metrics DTx^a^ evolves faster than trials Cross-cultural inconsistencies	Real-world data from EHRs^b^ and wearables Home-based cognitive training Global consensus on protocols Harmonized clinical trial methods
Regulatory uncertainty and policy gaps	No dedicated DTx regulatory pathway Inconsistent prescribing policies Delayed approvals Insufficient postmarket surveillance	Adaptive frameworks and sandboxes Unified criteria across regions Policy guidance (eg, AI+^c^ in China) Dynamic postmarket systems and policies
Patient acceptance and engagement	Low digital literacy Poor perceived value High dropout rates Interface usability issues Lack of provider support	User-centered and co-design development Simple, tailored interfaces Clinician endorsement Inclusive engagement strategies
Data privacy and ethical concerns	Data privacy vulnerabilities Complex consent processes Inconsistent global protection laws Exclusion of underserved groups	Stronger data protection (eg, GDPR)^d^ Transparent, simplified consent Algorithmic fairness Multistakeholder ethical governance
Data interoperability and system integration	Fragmented health IT^e^ systems Lack of standard APIs^f^ Limited infrastructure IoT^g^-EHR compatibility issues	HL7 FHIR^h^-based integration Blockchain-secured exchange Personalized decision support Interoperable digital ecosystems

a DTx: digital therapeutics.

b EHR: electronic health record.

c AI+: artificial intelligence plus (applications).

d GDPR: General Data Protection Regulation.

e IT: information technology.

f API: application programming interface.

g IoT: internet of things.

h FHIR: Fast Healthcare Interoperability Resources.

## Conclusion

Cognitive impairment presents a significant global health challenge, affecting millions of individuals and placing a substantial burden on patients, caregivers, and health care systems. CDTx have emerged as a promising innovation in the assessment, intervention, and management of cognitive disorders. However, several critical challenges remain, including the need for rigorous clinical validation, the establishment of comprehensive regulatory frameworks, and the enhancement of patient acceptance and engagement to support widespread adoption. Furthermore, data security and ethical issues, data interoperability, and system integration must be addressed to ensure the seamless integration of CDTx into clinical practice.

Despite these challenges, the potential of CDTx to transform the care and management of cognitive impairment is undeniable. Innovations in digital cognitive assessment, intervention, and management and monitoring technologies offer unprecedented opportunities for early detection and more efficient, patient-centered care in health care delivery. The rapid advancement of AI technologies, such as ChatGPT (OpenAI) and DeepSeek-R1 (DeepSeek), is expanding the role of DTx in cognitive health, enabling more personalized and scalable solutions.

Moving forward, a multistakeholder approach involving researchers, health care professionals, technology developers, policy makers, and regulators is essential. Future research should focus on developing standardized clinical validation methods, harmonizing regulatory frameworks globally, and enhancing patient and health care professional acceptability and engagement. Policy makers must prioritize the creation of supportive regulatory environments that balance innovation with patient safety while ensuring equitable access to these technologies. Clinically, integrating AI-driven tools into existing health care systems will require both technological advancements and operational adjustments to ensure effectiveness and scalability.

Ultimately, the integration of DTx into clinical practice for cognitive impairment holds significant potential to improve patient outcomes and to transform how cognitive health care is delivered, while also alleviating the burden on caregivers and health care systems worldwide. As AI continues to drive innovation, the development and scalable implementation of CDTx are poised to play a pivotal role in shaping the future of cognitive care. Coordinated, long-term, multistakeholder efforts will be essential to ensure their broad adoption and meaningful integration into routine clinical practice for cognitive impairment.

## Abbreviations

AD: Alzheimer disease
AI: artificial intelligence
CDTx: cognitive digital therapeutics
DTx: digital therapeutics
EEG: electroencephalography
EHR: electronic health record
EL: ensemble learning
FHIR: Fast Healthcare Interoperability Resources
MRI: magnetic resonance imaging
PAS: paired associative stimulation
VR: virtual reality
